# 

**Published:** 1908-03-07

**Authors:** 


### BOOKS RECEIVED.

Bale, Sons, and Danielsson, Ltd.

"The Pyonex; Its Theory and Practice." By W. B. Rule, M.R.C.S.

Health Resorts Bureau.

"Guide to Lake Gard." By J. Greble Leech.

Local Government Journal.

"Local Government Annual and Official Directory, 1908."

J. Wright and Co., Ltd., Bristol.

"First Aid to the Injured." 5th edition. By F. J. Warwick, B.A., M.B., and A. C. Tunstall, M.D.

Sidney Appleton.

"Text-Book of Treatment." Part I.: Obstetrics and Gynæcology. By W. Calwell, M.A., M.D., John Campbell, M.A., M.D., and Robert Campbell, B.A., M.B.

"Rummage." By H. C. P. Pattin.

The Caxton Publishing Co.

"The Edinburgh Stereoscopic Atlas of Obstetrics." Section I. Edited by G. F. Barbour Simpson, M.D., and Edward Burnet, B.A., M.B.

G. Routledge and Sons, Ltd.

"The Case for the Goat." By "Home Counties."

### THE GRADUATES' MEDICAL SCHOOLS.

### Diary for the week March 9 to 14.

### THE ROYAL COLLEGE OF SURGEONS.

**At 5 p.m.**

**March 9 and 11,** Prof. Ralph Thomson, **The Anatomy of the Long Bones, related to Certain Fractures.**

**March 13,** Prof. Sampson Handley, **The Natural Cure of Cancer.**

### ROYAL COLLEGE OF PHYSICIANS, Pall Mall East.

**At 5 p.m.**

**March 10 and 12,** Dr. J. W. H. Eyre, **Malta Fever.**

### LONDON SCHOOL OF CLINICAL MEDICINE, Seamen's Hospital, Greenwich.

**At 3.15 p.m.**

**March 9,** Mr. Wm. Turner, **Alveolar Abscess.**

**At 3.30 p.m.**

**March 11,** Mr. Cargill, **Paralytic Squint.**

### MOUNT VERNON HOSPITAL, Hampstead.

**At 5 p.m.**

**March 12,** Mr. Harold Barwell, **Cases of Laryngeal Disease.**

### NORTH-EAST LONDON POST-GRADUATE COLLEGE, Prince of Wales's General Hospital, Tottenham, N.

**At 4.30 p.m.**

**March 12,** Dr. A. J. Whiting, **Angioneurotic Œdema.**

### HOSPITAL FOR CONSUMPTION AND DISEASES OF THE CHEST, Brompton, S.W.

**At 4 p.m.**

**March 11,** Dr. Dundas Grant, **Demonstration of Cases from the Throat Department.**

### THE POST-GRADUATE COLLEGE, West London Hospital, Hammersmith, W.

**At noon.**

**March 9,** Dr. Low, **Pathological Demonstration.**

**March 10 and 12,** Dr. Pritchard, **Practical Medicine.**

**At 5 p.m.**

**March 9 and 11,** Mr. Armour, **Clinical—I. and II.**

**March 10,** Dr. Pritchard, **On the Vaccination Treatment of Infective Diseases.—II.**

**March 12,** Mr. Baldwin, **Practical Surgery—V.**

**March 13,** Mr. R. W. Lloyd, **Anæsthetics—II.**

### CENTRAL LONDON THROAT and EAR HOSPITAL, Gray's Inn Road, W.C.

**At 4 p.m.**

**March 10 and 13,** Dr. Dundas Grant, **Methods of Examination.**

**March 12,** Dr. Andrew Wylie, **Tinnitus.**

**March 12,** Mr. Stuart Low, **Cancer of the Throat.**

### MEDICAL GRADUATES' COLLEGE AND POLYCLINIC, Chenies Street, W.C.

**At 5.15 p.m.**

**March 9,** Mr. Bishop Harman, **Contusions of the Eyeball.**

**March 10,** Dr. F. Langmead, **Some Modern Methods of Infant Feeding.**

**March 11,** Mr. A. H. Tubby, **Surgical Diseases of Children.**

**March 12,** Dr. Charles Mercier, **Functional Disease and its Treatment.**

### NATIONAL HOSPITAL FOR THE PARALYSED AND EPILEPTIC, Queen Square, Bloomsbury, W.C.

**At 3.30 p.m.**

**March 10,** Dr. Batten, **Subacute Combined Degeneration of the Spinal Cord.**

**March 13,** Mr. Paton, **Ocular Paralysis.**

### CHARING CROSS HOSPITAL, W.C.

Post-Graduate Course

**At 4 p.m.**

**March 12,** Dr. Forsyth, **Medical.**

### THE HOSPITAL FOR SICK CHILDREN, Great Ormond Street, W.C.

**At 4 p.m.**

**March 12,** Mr. Arbuthnot Lane, **Fractures in Children.**

### THE HOSPITAL FOR NERVOUS DISEASES, Welbeck Street, W.

**At 5 p.m.**

**March 12,** Dr. T. D. Savill, **The Psychology of Hysteria.**

The rate of annual subscriptions to The Hospital, either direct from the Office or through agents, is :—

For the United Kingdom... ... ... 15s. a year
To the Colonies and Abroad ... ... 17s. a year inclusive of postage in each case.

THE HOSPITAL *March* 7, 1908.

*Name* ..........................................................................

*Address* ......................................................................

.......................................................................................

This Coupon must accompany manuscript or contributions intended for The Hospital.

